# Constructing Dynamic Brain Functional Networks via Hyper-Graph Manifold Regularization for Mild Cognitive Impairment Classification

**DOI:** 10.3389/fnins.2021.669345

**Published:** 2021-04-01

**Authors:** Yixin Ji, Yutao Zhang, Haifeng Shi, Zhuqing Jiao, Shui-Hua Wang, Chuang Wang

**Affiliations:** ^1^School of Microelectronics and Control Engineering, Changzhou University, Changzhou, China; ^2^School of Computer Science and Artificial Intelligence, Changzhou University, Changzhou, China; ^3^Department of Radiology, Changzhou Second People’s Hospital Affiliated to Nanjing Medical University, Changzhou, China; ^4^School of Informatics, University of Leicester, Leicester, United Kingdom; ^5^School of Medicine, Ningbo University, Ningbo, China

**Keywords:** mild cognitive impairment, Alzheimer’s disease, dynamic brain functional network, manifold regularization, hyper-graph

## Abstract

Brain functional networks (BFNs) constructed via manifold regularization (MR) have emerged as a powerful tool in finding new biomarkers for brain disease diagnosis. However, they only describe the pair-wise relationship between two brain regions, and cannot describe the functional interaction between multiple brain regions, or the high-order relationship, well. To solve this issue, we propose a method to construct dynamic BFNs (DBFNs) via hyper-graph MR (HMR) and employ it to classify mild cognitive impairment (MCI) subjects. First, we construct DBFNs via *Pearson*’s correlation (PC) method and remodel the PC method as an optimization model. Then, we use *k*-nearest neighbor (KNN) algorithm to construct the hyper-graph and obtain the hyper-graph manifold regularizer based on the hyper-graph. We introduce the hyper-graph manifold regularizer and the *L*1-norm regularizer into the PC-based optimization model to optimize DBFNs and obtain the final sparse DBFNs (SDBFNs). Finally, we conduct classification experiments to classify MCI subjects from normal subjects to verify the effectiveness of our method. Experimental results show that the proposed method achieves better classification performance compared with other state-of-the-art methods, and the classification accuracy (ACC), the sensitivity (SEN), the specificity (SPE), and the area under the curve (AUC) reach 82.4946 ± 0.2827%, 77.2473 ± 0.5747%, 87.7419 ± 0.2286%, and 0.9021 ± 0.0007, respectively. This method expands the MR method and DBFNs with more biological significance. It can effectively improve the classification performance of DBFNs for MCI, and has certain reference value for the research and auxiliary diagnosis of Alzheimer’s disease (AD).

## Introduction

Alzheimer’s disease (AD) is a primary degenerative brain disease that occurs in senectitude and presenium (Lu et al., 2019; Bi et al., 2021). AD creates issues in memory, thinking, analysis, judgment, visual and spatial recognition, and emotional regulation. However, there are currently no specific treatments or therapeutic drugs to reverse disease progression. Mild cognitive impairment (MCI) is also a type of dementia, and is an intermediate stage between normal people and AD patients. In clinical practice, MCI is mostly manifested as a decline in cognitive function and memory, but it does not affect the daily life of patients (Muldoon and Bassett, 2016). Related research has shown that the annual conversion rate of MCI to AD is about 10–15% (Jiao et al., 2014; Zhang et al., 2015b). MCI due to AD provides a potential window to detect and diagnose AD before significant neurodegeneration has begun. Early active intervention treatment for MCI can improve or delay its cognitive decline and even the development of AD (Alzheimer’s Association, 2012). Therefore, the accurate identification of MCI and the intervention of MCI through drug and non-drug pathways to reduce the AD conversion rate have attracted great attention from researchers (Gauthier et al., 2006; Tobia et al., 2017). It is important to explore which subjects will progress from MCI to AD, as there are predictors of progression that will indicate a more rapid rate of progression in MCI subjects.

Nowadays, neuroimaging technology is widely used in the detection and research of brain diseases. Some existing brain imaging techniques include magnetic resonance imaging (MRI) technology (Zhang et al., 2015a), functional MRI (fMRI) (Zhang Y. D. et al., 2016), and diffusion MRI (Basser and Pierpaoli, 2011). Electrophysiology techniques, including electroencephalogram (EEG) (Jung et al., 2000), magnetoencephalography (MEG) (Smythies et al., 2005), and positron emission technology (PET) (Mourik et al., 2009), provide effective and non-invasive methods to explore the brain and its connection patterns, revealing brain functions and brain structures that could not be revealed before. Many medical and biological studies have shown that human cognitive processes usually rely on pair-wise relationships between different neurons and brain regions (Ou et al., 2015). The brain functional network (BFN) can describe the function or structural interaction of the brain at the entire brain connection level (Rubinov and Sporns, 2010); thus, it provides a new tool for exploring the function and structure of the brain. In the research based on resting-state fMRI, the BFN is generally constructed through the full time series of resting state. Most recent studies have shown that brain neural activity changes dynamically over time, and this dynamic change will contain more abundant information (Chang and Glover, 2010). Therefore, research on dynamic BFN (DBFN) will help us further explore the operation mode of the whole brain, and it is conducive to the auxiliary diagnosis of brain diseases.

In research based on BFNs, how to construct BFNs is a very important procedure. Researchers have proposed many methods for constructing BFNs, from the simplest method for constructing BFNs based on *Pearson*’s correlation (PC) (Jiang et al., 2019), to the partial correlation method (Jiang et al., 2019), to the dynamic causal model method (Roebroeck et al., 2005), etc. However, these methods have their shortcomings. For example, the PC method can only calculate the full correlation, and it cannot remove the redundant effects of other brain regions. The BFN construction method based on partial correlation may lead to ill-posed problems (Li et al., 2019). Now, adding regularizers to the PC method or the partial method can result in better BFNs. Regularizers mainly reflect some prior information of the brain, such as sparsity (Qiao et al., 2016), modularity (Qiao et al., 2016), group sparsity (Wee et al., 2014), scale-free property (Li et al., 2017), etc. These properties are transformed into corresponding regularizers embedded in the construction of BFNs through certain transformations to obtain BFNs containing more prior information.

Recently, BFNs via manifold regularization (MR) have been widely used in studies. About MR, Li et al. (2020c) proposed a hypothesis: if two brain regions are very close in space, then the functional connections between them and other brain regions may share similar connection patterns. It means that these brain regions have similar topological properties. Li et al. (2020c) transformed this similarity into a manifold regularizer and introduced it to construct BFNs. Xue et al. (2020) constructed BFNs based on the same idea, and introduced the distance information between brain regions into the manifold regularizers. However, most studies just consider the pair correlation between brain regions, but ignore the high-order relationship which reflects interactive information between multiple brain regions. This could be a drawback because the BFN itself is a complex network. Recent studies have shown that a brain region usually directly interacts with several neighboring brain regions, forming a complex interactive relationship. Therefore, the high-order relationship between brain regions may contain some discriminative information to improve the classification performance. Hyper-graph is a good choice to describe the high-order relationship between multiple nodes in a graph (Yu et al., 2014), and has been successfully applied in many fields. In traditional graphs, one edge of the graph can only connect two related vertices. In practice, the relationship between objects is much more complicated than the pairwise relationship. Hyper-graph is an extension of traditional graphs. In a hyper-graph, a hyper-edge is a collection of any number of nodes, which can connect any number of nodes, so it is natural to use hyper-graphs to model high-order relationships. Zhou et al. (2007) proposed a hyper-graph learning method for clustering, classification, and embedding learning, and the hyper-graph Laplacian operator was used to describe the complex relationship between multiple samples. Jie et al. (2016) used sparse representation (SR) method to construct hyper-graph and applied it to the diagnosis of AD and MCI patients.

Most of the above studies performed feature extraction, feature selection, and classification for hyper-graph directly. But few studies convert the hyper-graph into a regularizer and introduce it into the construction of BFNs. To solve these problems, we propose a method for constructing DBFNs via hyper-graph MR (HMR) and apply this method to differentiate MCI subjects from normal subjects. First, we construct DBFNs and transform the PC method into an optimization model. Next, we construct hyper-graphs based on DBFNs and obtain the hyper-graph manifold regularizer. Then, we introduce the hyper-graph manifold regularizer and *L*1-norm regularizer into the optimization model of the PC method to obtain the sparse DBFNs (SDBFNs). After that, we extract the weighted-graph local clustering coefficient of each brain region in two types of subjects’ SDBFNs as an effective feature and use *t*-test for feature selection from SDBFNs. Finally, we train a linear kernel support vector machine (SVM) to classify the SDBFNs of all subjects and analyze the classification performance. Furthermore, we also investigate the parameter sensitivities on classification performance and some discriminative brain regions.

## Materials and Methods

### Data Acquisition and Processing

The subjects were recruited through local newspapers and media in North Carolina^1^ (Qiao et al., 2016; Li et al., 2020b). They are all right-handed and have no history of neurological or mental illness, and no history of alcohol or drug abuse. Excluding these who frequently use psychotropic drugs, stimulants, and β-blockers, all subjects received standard neuropsychological assessments and responses.

Raw fMRI images are scanned by the 3T Siemens TRIO scanner. The image size is 74 × 74 × 45, the voxel size is 2.97 × 2.97 × 3 mm^3^, and the repetition time (TR) is 3000 ms with 180 volumes. The raw resting-state fMRI data are preprocessed by using the SPM toolbox^2^ and DPARSFA^3^ toolbox of Matlab R2012a software. In order to avoid signals dithering, the first 10 fMRI images are discarded. The remaining images are first corrected in time layer and head motion, and then the images are spatially normalized and linear drift removed. Band-pass filtering is performed with 0.01–0.08 Hz to remove the interference of blood flow and power frequency. In addition, the generalized linear model is used to remove covariates such as head movement parameters, white matter, gray matter, and cerebrospinal fluid. Finally, we clean the data with frame-wise displacements (FD) > 0.5. Data are registered through the Anatomical Automatic Labeling (AAL) atlas (Tzourio-Mazoyer et al., 2002), and blood oxygenation level-dependent (BOLD) signals in each brain region are extracted by means of mean value. Screened by data time points are greater than 80, and BOLD signals of 91 subjects (45 MCI subjects and 46 normal subjects) are retained. Table 1 shows the specific group characteristics of the subjects, including their Mini-Mental State Examination (MMSE) scores.

**TABLE 1:** The specific group characteristics of the subjects.

**Group characteristic**	**MCI**	**Normal**
Gender (male/female)	25M / 20F	14M / 32F
Age (mean ± SD)	74.13 ± 6.68	73.5 ± 3.50
MMSE (mean ± SD)	27.71 ± 1.73	28.10 ± 1.35

### Conventional DBFN Construction

Suppose **X** = [**x**_1_,**x**_2_,…,**x**_*p*_] ∈ *ℝ*^*Q*×*P*^ is a time series matrix, *Q* is the total number of time points, *P* is the number of brain regions, and **x**_*i*_,**x**_*j*_ ∈ *ℝ*^*Q*×1^ are the time series vectors of the *i*th brain region and the *j*th brain region. We use the sliding window method to divide the entire time series into several overlapping time sub-segments (Chen et al., 2016). Assuming that the window width is *N* and the step size is *S*, defining ${\text{x}}_{i}^{\left(l\right)}\,\left(k\right)\in {\mathbb{R}}^{N\times 1}$ as the *k*-th sub-segment extracted from the time series of the *l*th subject. The total number of windows *K* is expressed as:

(1) $$K=\frac{Q-N}{S}+1,1\leq k\leq K$$

Then we calculate the PC coefficient between each sub-segment and construct DBFNs. ${\text{x}}_{i}^{k}\in {\mathbb{R}}^{N},\left(k=1,\ldots ,K\right)$ denotes the time series of the *i*th brain region in the *k*th window, and the time series matrix ${\text{X}}^{\left(k\right)}=\left[{\text{x}}_{1}^{k},{\text{x}}_{2}^{k},\ldots ,{\text{x}}_{P}^{k}\right]\in {\mathbb{R}}^{N\times P}$ in the *k*th window concatenate ${\text{x}}_{i}^{k}$ in series. The correlation coefficient matrix of BFN *W*^(^*^*k*^*^)^ in the *k*th window is **W**^(*k*)^ = (**X**^(*k*)^)^*T*^**X**^(*k*)^. Convert this formula to the optimized form as:

(2) minW⁢(k)⁢||W(k)-X(k)T⁢X(k)||F2

### BFN Construction Based on MR

Li et al. (2020c) were inspired by the existence of similar connection patterns (i.e., similar internal structures) in BFNs and proposed a method for constructing sparse BFNs via MR. Li et al. (2020c) also extended MR, embedded the sparse prior information, and obtained the extended method SMR. The objective function of SMR can be formulated as:

(3) $$\min_{W}\,{||\text{X-XW}||}_{F}^{2}+\lambda \,{||W||}_{1}+\beta \,t\,r\,\left({\text{W}}^{T}\,\text{LW}\right)$$

where $||.|{|}_{F}^{2}$ represents the square of the *F*-norm, ||.||_1_ represents the *L*1-norm, *λ* is a regularization parameter of *L*1-norm regularizer, and β is the regularization parameter of manifold regularizer. tr(.) represents the trace of the matrix, ***L*** is the Laplacian matrix, and its solution method is $\text{L}=\text{I}-{\text{D}}^{-\frac{1}{2}}\,{\text{SD}}^{-\frac{1}{2}}$. ***I*** is the identity matrix and ***D*** is a diagonal matrix. The diagonal elements in ***D*** are expressed as $D_{i\,i}=\sum_{j=1}^{N}W_{i\,j}$. ***S*** is the correlation coefficient matrix of the BFN constructed based on the PC method. When *λ* = 0, this method changes into the BFN construction method based on MR.

### DBFN Construction Based on HMR

Hyper-graph is an extension of conventional graph. Denote a hyper-graph as ***G*** (***V***, ***E***, ***A***), where ***V*** represents the set of vertices, ***E*** represents the set of hyper-edges, and ***A*** represents the set of weights of each hyper-edge. For the hyper-graph ***G***, we use the correlation matrix **H** ∈ *ℝ*^|**V**|×|**E**|^ to describe the relationship between vertices and hyper-edges; it can be formulated as:

(4) $$H\,\left(v,e\right)=\left\{ \begin{array}{c} 1,i\,f\,\,v\in e\\ 0,i\,f\,\,v\notin e \end{array}\right.\,$$

where *v* ∈ **V** is a node in ***G*** and *e* ∈ **E** is a hyper-edge in ***G***.

For the correlation matrix ***H***, the node degree of each node and the edge degree of each hyper-edge can be formulated as:

(5) $$d\,\left(v\right)=\sum_{e\in E}a\,\left(e_{b}\right)\,\,h\,\left(v,e_{b}\right),\,b=1,\ldots ,M$$

(6) $$\delta \,\left(e_{b}\right)=\sum_{v\in V}h\,\left(v,e_{b}\right),\,b=1,\ldots ,M$$

where *e*_*b*_ (*b* = 1,…, *M* and *M* represents the number of hyper-edges) represents the *b*th hyper-edge and *a*(*e*_*b*_) represents the weight of *e*_*b*_. MR explores the internal geometric structure of the graph by means of the Laplacian matrix. Similarly, the Laplacian matrix of the hyper-graph can better reflect the high-order relationship between multiple samples for HMR. Many methods of calculating the Laplacian matrix of the hyper-graph can be roughly divided into two categories: one category is to construct a simple graph based on the original hyper-graph, and then calculate the Laplacian matrix on the simple graph (Zien et al., 1999); another category is to directly derive the Laplacian matrix of the hyper-graph based on the Laplacian matrix of the simple graph (Zhou et al., 2007). By comparison, we use the second method to calculate the Laplacian matrix of the hyper-graph:

(7) $${\text{L}}^{h}=\text{I}-\Theta$$

where ***L****^*h*^* is the Laplacian matrix of the hyper-graph, ***I*** is the identity matrix, and $\Theta ={\text{D}}_{v}^{-\frac{1}{2}}\,{\text{HAD}}_{e}^{-1}\,{\text{H}}^{T}\,{\text{D}}_{v}^{-\frac{1}{2}}$, ***D****v* represents the diagonal matrix and its diagonal elements are *d*(*v*), and ***D****e* represents the diagonal matrix and its diagonal elements are δ(*e*_*b*_). ***A*** represents the diagonal matrix and its diagonal elements are hyper-edge weights. Referring to the methods of Zhou et al. (2007) and Shao et al. (2019), we adopt *k*-nearest neighbor (KNN) algorithm to construct the hyper-graph based on DBFNs.

Inspired by the research of Li et al. (2017), we propose a method for constructing DBFNs based on HMR, and add the *L*1-norm regularizer based on HMR, and obtain a new DBFN construction method, namely, SHMR. The objective function of SHMR is as follows:

minW(k)⁢||W(k)-X(k)T⁢X(k)||F2+λ⁢||W(k)||1

(8) $$+\text{β}tr\left(W^{\left(k\right)}{}^{\text{T}}L^{h}W^{\left(k\right)}\right)$$

where ***X***^(^*^*k*^*^)^ represents the time series matrix of the *k*th window, *λ* represents the regularization parameter of *L*1-norm, and β represents the regularization parameter of manifold regularizer. When *λ* = 0, the method changes into the DBFN construction method based on HMR.

In Formula (8), the derivable part is the fitting term and the manifold regularizer and the non-derivable part is the L1-norm regularizer. We use the proximal operator method (Yan et al., 2013) to optimize and solve the non-derivable part. Then the gradient of the fitting term f=||W(k)-X(k)T⁢X(k)||F2 is calculated as:

(9) ∇W(k)⁡f⁢(X(k),W(k))=2⁢(W(k)-X(k)T⁢X(k))

Then we update ***W***^(^*^*k*^*^)^ *m* times:

(10) $${\text{W}}_{m}^{\left(k\right)}={\text{W}}_{m-1}^{\left(k\right)}-\alpha_{m}\,\left(\nabla_{W^{\left(k\right)}}f\,\left({\text{X}}^{\left(k\right)},{\text{W}}_{m-1}^{\left(k\right)}\right)+\beta \,{\text{L}}^{h}\,{\text{W}}^{\left(k\right)}\right)$$

where α*_*m*_* represents the step size in gradient descent.

Then we calculate the proximal operator of the L1-norm regularizer which can be formulated as:

(11) $$proximal_{\lambda ||.|{|}_{1}}\left({\text{W}}_{m}^{\left(k\right)}\right)=[sgn\left(W_{i\,j}^{\left(k\right)}\right)$$

$$\times max\left(abs\left(W_{ij}^{\left(k\right)}\right)-\text{λ}\right),{\left.0\right]}_{N\times N}$$

The intention of Formula (11) is to apply a soft threshold operation to the elements in ${\text{W}}_{m}^{\left(k\right)}$. After each gradient descent calculation is completed, we use the proximal operator to solve the constraint of ***W***^(^*^*k*^*^)^.

Accordingly, we adopt the same strategy as in the study of Elhamifar and Vidal (2013) and symmetrize ***W***^(^*^*k*^*^)^; finally, we obtain ${\text{W}}^{{*}^{\left(k\right)}}=\frac{W^{\left(k\right)}+W^{{\left(k\right)}^{T}}}{2}$. We use W^*^(*k*)^^to represent the DBFN constructed by SHMR, namely, SDBFN.

### Feature Extraction, Feature Selection, and Classification via SDBFN

The weighted-graph local clustering coefficient has been widely used in the analysis of BFN, and related studies have also shown that the clustering properties of BFN have changed in neurological diseases (such as AD and MCI) (Jiao et al., 2019). Giving a network of *N* nodes, the weighted-graph local clustering coefficient of node *i* can be formulated as:

(12) $$C_{i}=\frac{2\,\sum_{i,j\in v_{i}}{\left(\omega_{i\,j}\right)}^{\frac{1}{3}}}{\left|{\text{v}}_{i}\right|\,\left(\left|{\text{v}}_{i}\right|-1\right)}$$

where ω_*ij*_ represents the weight of the connection edge between node *i* and node *j*, ***v****_*i*_* represents the set of nodes directly connected to node *i*, and |***v****_*i*_*| represents the number of elements in ***v****_*i*_*.

The generalization ability of SVM is excellent, and the process of transformation from non-linear problem to linear problem can be realized by kernel function. SVM solves the local optimal problem and curse of dimensionality problem in small sample non-linear space. In order to avoid the confusing effect of feature extraction and the selection of the classifier on the classification performance, we calculate the weighted-graph local clustering coefficients in SDBFNs as effective features and use the *t*-test method for feature selection, and finally we train a linear kernel SVM to classify the SDBFNs of all subjects. We use four metrics to evaluate the classification performance: accuracy (ACC), sensitivity (SEN), specificity (SPE), and area under the curve (AUC) (Li et al., 2018).

## Experimental Results

### Parameter Sensitivity on Classification Performance

In this section, we discuss the sensitivities of different parameters on MCI classification performance. Since there are multiple parameters in our method, the grid search method cannot be used directly to find the optimal parameter. Our strategy is to find the optimal parameter separately, that is, to find each optimal parameter step by step.

#### Sensitivity of Different Window Width and Step Size

The window width *S* and step size *V* have an important influence on constructing DBFNs and SDBFNs. Since SDBFN is optimized based on DBFN, we first classify DBFN of all subjects based on different window widths and step sizes to determine the optimal window width and step size. The specific process of classification is as follows. First, we extract the weighted-graph local clustering coefficients in DBFNs of all subjects, which are constructed with different window widths and step sizes. Then we use the *t*-test method for feature selection, with the significance level of 0.05. Finally, we choose linear kernel SVM classifier to classify all subjects, and the linear kernel SVM classifier is implemented using the LIBSVM toolbox (Chang and Lin, 2011). In classification, MCI subjects are generally regarded as positive samples, and normal subjects are regarded as negative samples. We use ACC, SEN, SPE, and AUC to measure the classification performance of different methods, and we also use 10-fold cross validation to verify the classification results (Li et al., 2020a; Xu et al., 2020) by taking the mean value of each classification index after 10 times of 10-fold cross-validation as the final results. We analyze the classification performance of multiple groups of window widths and step sizes to find the optimal parameter. The classification performance of different window widths and step sizes and the standard deviation (STD) of each index are shown in Table 2. The best classification performance is highlighted in black. Among them, the step size varies from 1 to 2 with an interval of 1 and the window width varies from 50 to 80 with an interval of 10.

**TABLE 2:** Classification performance of different window widths and step sizes.

**Method**	**ACC (%) ± STD**	**SEN (%) ± STD**	**SPE (%) ± STD**	**AUC ± STD**
*V* = 1,*S* = 50	**81.0570 ± 0.2551**	**77.2975 ± 0.3529**	86.4165 ± 0.3682	0.8986 ± 0.0026
*V* = 2,*S* = 50	76.4275 ± 0.4389	66.9583 ± 0.5456	85.8976 ± 0.5863	0.8409 ± 0.0023
*V* = 1,*S* = 60	80.7219 ± 0.4070	71.1746 ± 0.4867	**90.2692 ± 0.4831**	0.9025 ± 0.0021
*V* = 2,*S* = 60	73.4818 ± 0.7597	59.4141 ± 1.0188	87.5494 ± 0.8488	0.8191 ± 0.0039
*V* = 1,*S* = 70	77.0553 ± 0.6533	66.3636 ± 1.1438	87.7470 ± 1.1023	0.8409 ± 0.0059
*V* = 2,*S* = 70	65.7299 ± 0.8631	43.8148 ± 1.7895	87.6449 ± 0.5250	0.7483 ± 0.0062
*V* = 1,*S* = 80	48.7391 ± 4.2414	14.0000 ± 3.1514	83.4783 ± 7.6827	0.5307 ± 0.0493
*V* = 2,*S* = 80	47.2440 ± 2.4217	16.4444 ± 2.3888	78.0435 ± 3.8956	0.5053 ± 0.0221

From Table 2, we can see that the ACC and SEN are better when the window length is 50 and the step size is 1. As the window width and step size increase, the classification performance becomes worse gradually. This is consistent with the conclusions in the research of Jiao et al. (2019) and Li et al. (2018). The reason may be that using a larger window width and larger step size will ignore the functional connections between some brain regions and part of the dynamic information that changes over time, so that the classification performance starts to decrease.

#### Sensitivity of the Number of Neighbors

We use the KNN algorithm to construct the hyper-graph. The specific process is to use the KNN algorithm to select the *k* nearest vertices to the center vertex to form a hyper-edge. The classification results of different neighbor numbers are shown in Table 3, and the values of *k* are set as 1, 3, 5, 7, 8, 9, 10, and 15 (Shao et al., 2019). When *k* = 1, it does not construct a hyper-graph. We can find that ACC, SEN, SPE, and AUC are the best when the value of *k* is 7, which is consistent with the conclusion in the study of Shao et al. (2019). When the value of *k* is larger than 7, the classification performance begins to decline. The possible reason for this is that when the value of *k* is larger, it describes the global structure information of the sample rather than the local distribution information. When the value of *k* is larger, the hyper-edge may contain many different types of samples, so it cannot reflect the real data structure well. In addition, when *k* = 1, the classification performance is slightly lower, indicating that the introduction of hyper-graph helps to improve the classification performance.

**TABLE 3:** Classification performance of different neighbor numbers.

**Method**	**ACC (%) ± STD**	**SEN (%) ± STD**	**SPE (%) ± STD**	**AUC ± STD**
*k* = 1	81.5969 ± 0.2353	77.2330 ± 0.2969	85.9607 ± 0.4100	0.8984 ± 0.0018
*k* = 3	82.1424 ± 0.3034	76.7742 ± 0.3528	87.5105 ± 0.4055	0.8988 ± 0.0011
*k* = 5	82.2696 ± 0.2158	77.1900 ± 0.4619	87.3492 ± 0.2770	0.8984 ± 0.0010
*k* = 6	81.9266 ± 0.3825	76.2796 ± 0.6879	87.5736 ± 0.2972	0.9001 ± 0.0015
*k* = 7	**82.4946 ± 0.2827**	**77.2473 ± 0.5747**	**87.7419 ± 0.2286**	**0.9021 ± 0.0007**
*k* = 8	82.2076 ± 0.1873	76.9677 ± 0.4484	87.4474 ± 0.2582	0.9003 ± 0.0013
*k* = 9	81.9021 ± 0.2479	76.2867 ± 0.3729	87.5175 ± 0.2706	0.8997 ± 0.0015
*k* = 10	81.6502 ± 0.3456	76.0143 ± 0.3929	87.2861 ± 0.4948	0.8977 ± 0.0014
*k* = 15	81.5525 ± 0.2362	75.7348 ± 0.2989	87.3703 ± 0.2506	0.8954 ± 0.0011

#### Sensitivity of Regularization Parameters

The role of L1-norm regularizer is mainly to remove redundant features and make DBFNs sparser. The hyper-graph manifold regularizer retains the discriminative information of each subject, thereby inducing more discriminative features. The regularization parameters λ and β are used to adjust the complexity of constructing DBFNs. We test the values of various classification indices for Normal and MCI subjects under different regularization parameters. The classification performance of SDBFNs obtained by different regularization parameters are shown in Figure 2, and the specific results are shown in Table 4, where the ranges of λ and β are both {2^–4^,2^–3^,2^–2^,2^–1^}.

**FIGURE 1:**
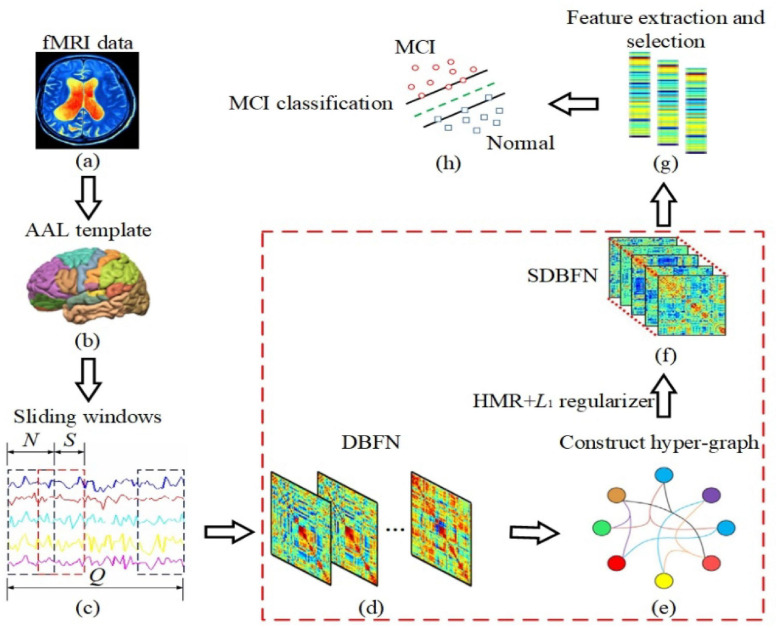
The framework of constructing SDBFNs via SHMR for MCI classification. The area marked in red box is the key research part. **(a)** Preprocessing the obtained resting-state fMRI data of two types of subjects; **(b)** registering the preprocessed resting-state fMRI data to 90 brain regions according to the AAL template, and obtaining the time series of all brain regions; **(c)** dividing the entire time series into multiple overlapping sub-sequence segments by sliding window method; **(d)** constructing DBFNs based on the PC method and transforming it into an optimized model; **(e)** constructing hyper-graphs based on DBFNs and obtaining hyper-graph Laplacian matrices; **(f)** constructing the manifold regularizer by hyper-graph Laplacian matrices, and introducing the manifold regularizer and *L*1-norm regularizer into the optimization model of the PC method to obtain SDBFNs; **(g)** extracting the weighted-graph local clustering coefficient of each brain region in SDBFNs, and using the *t*-test for feature selection; and **(h)** training a linear kernel SVM classifier to classify the SDBFNs of all subjects and analyzing the classification performance.

**FIGURE 2:**
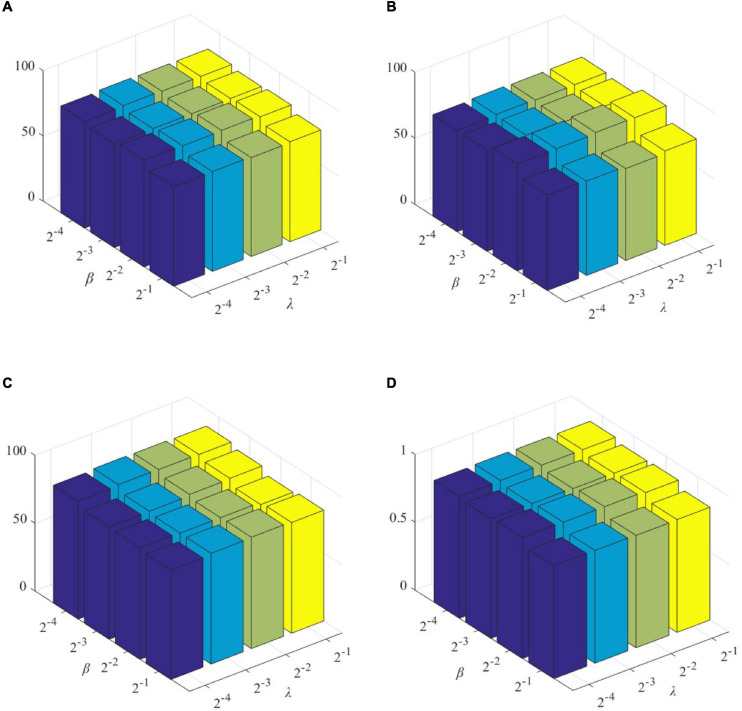
Classification performance of SDBFNs obtained by different regularization parameters: **(A)** ACC, **(B)** SEN, **(C)** SPE, and **(D)** AUC.

**TABLE 4:** Classification performance of different regularization parameter values.

**Method**	**ACC (%) ± STD**	**SEN (%) ± STD**	**SPE (%) ± STD**	**AUC ± STD**
λ = 2^–4^, β = 2^–4^	81.7265 ± 0.2902	76.5806 ± 0.3397	86.8724 ± 0.4542	0.8980 ± 0.0013
λ = 2^–4^, β = 2^–3^	**82.4946 ± 0.2827**	77.2473 ± 0.5747	**87.7419 ± 0.2286**	**0.9021 ± 0.0007**
λ = 2^–4^, β = 2^–2^	82.1426 ± 0.2041	76.7957 ± 0.2552	87.4895 ± 0.2789	0.9008 ± 0.0018
λ = 2^–4^, β = 2^–1^	82.1063 ± 0.2744	77.0036 ± 0.4272	87.2090 ± 0.2886	0.9016 ± 0.0007
λ = 2^–3^, β = 2^–4^	79.5965 ± 0.3622	76.4301 ± 0.3820	82.7630 ± 0.5875	0.8818 ± 0.0022
λ = 2^–3^, β = 2^–3^	79.7052 ± 0.4198	76.4229 ± 0.7407	82.9874 ± 0.5474	0.8796 ± 0.0021
λ = 2^–3^, β = 2^–2^	79.7154 ± 0.3038	75.4265 ± 0.4231	84.0042 ± 0.4187	0.8770 ± 0.0024
λ = 2^–3^, β = 2^–1^	80.0542 ± 0.2779	75.6344 ± 0.5218	84.4741 ± 0.5414	0.8768 ± 0.0027
λ = 2^–2^, β = 2^–4^	81.5949 ± 0.3644	80.9247 ± 0.6929	82.2651 ± 0.4942	0.8930 ± 0.0018
λ = 2^–2^, β = 2^–3^	81.3470 ± 0.2691	81.0251 ± 0.3718	81.6690 ± 0.7053	0.8910 ± 0.0019
λ = 2^–2^, β = 2^–2^	81.7879 ± 0.2006	**81.9068 ± 0.4611**	81.6690 ± 0.3105	0.8918 ± 0.0014
λ = 2^–2^, β = 2^–1^	80.8248 ± 0.5051	81.6918 ± 0.5472	79.9579 ± 0.7180	0.8821 ± 0.0037
λ = 2^–1^, β = 2^–4^	76.2415 ± 0.2577	71.9570 ± 0.2883	80.5259 ± 0.4664	0.8321 ± 0.0015
λ = 2^–1^, β = 2^–3^	76.4409 ± 0.3330	71.2688 ± 0.3996	81.6129 ± 0.4470	0.8312 ± 0.0021
λ = 2^–1^, β = 2^–2^	75.7620 ± 0.4170	69.4552 ± 0.5639	82.0687 ± 0.5143	0.8283 ± 0.0017
λ = 2^–1^, β = 2^–1^	76.4985 ± 0.2997	71.4122 ± 0.3636	81.5849 ± 0.3956	0.8313 ± 0.0018

From Figure 2 and Table 4, we can find that the ACC, SEN, SPE, and AUC are best when λ = 2^–4^ and β = 2^–3^. With the increase of λ and β, the classification performance starts to decrease. According to the above experiments, we set the window width to 50, the step size to 1, the number of neighbors to 7, and λ = 2^–4^ and β = 2^–3^ to construct SDBFNs.

### Visualization of BFNs

**FIGURE 3:**
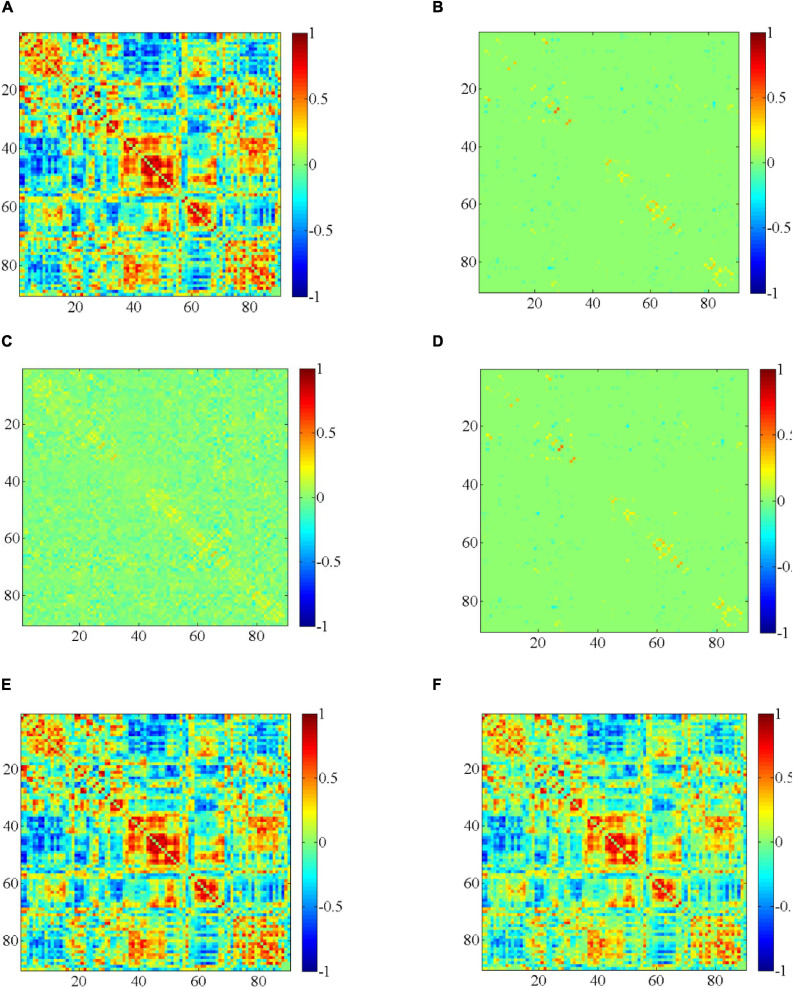
Visualization results of constructing the BFN in the same time window by different methods. **(A)** PC, **(B)** SR, **(C)** MR, **(D)** SMR, **(E)** HMR, and **(F)** SHMR.

We randomly select a subject, then we use different methods to construct DBFNs, and visualize the BFN in the same time window. These comparison methods are related to our method, as shown in Figure 3. The compared methods that we employ include the PC method (Jiang et al., 2019), the SR method (the regularization parameter corresponding to the optimal classification performance is 2^4^) (Jiang et al., 2019), the MR method (the regularization parameter corresponding to the optimal classification performance is 2^–4^) (Li et al., 2020c), the SMR method (the regularization parameters corresponding to the optimal classification performance are 2^4^ and 2^–1^), and the HMR method (the regularization parameter corresponding to the optimal classification performance is 2^–3^). Figures 3A–F are the visualized results of constructing the BFN in the same time window by different methods.

Figure 3 shows the visualization results of constructing the BFN in the same time window by different methods. From these visualization results, we can find that the BFN constructed based on the PC method in the same time window is often dense, while the BFN constructed based on the SR method in the same time window is sparse. Figure 3D is sparser than Figure 3A and the topological structure is clearer, while Figures 3B,C have stronger functional connection strength.

### Classification Performance for MCI by Different Methods

We compare the classification performance of different DBFN construction methods for MCI identification, where the best classification performance is highlighted. As shown in Table 5, the classification performance of SHMR for MCI is better than other methods, expect SEN. In particular, its ACC, SEN, SPE, and AUC are 82.4946 ± 0.2827%, 77.2473 ± 0.5747%, 87.7419 ± 0.2286%, and 0.9021 ± 0.0007, respectively. The best classification performance among the compared methods is the HMR method, and its ACC, SEN, SPE, and AUC are 81.4570 ± 0.2727%, 76.6237 ± 0.3087%, 86.2903 ± 0.3670%, and 0.9005 ± 0.0017, respectively. The classification performance of the SMR method is better than that of the SR method, but the classification performance of MR is worse than that of the SR method. It shows that the simultaneous introduction of L1-norm regularizer and manifold regularizer based on the SR method can effectively improve the quality of DBFNs and enhance the classification ACC effectively, while the introduction of L1-norm regularizer alone cannot improve the classification performance. This result is similar to the research of Li et al. (2020c). The classification performances of the SHMR method and the HMR method are all better than that of the PC method; it indicates the effectiveness of introducing the hyper-graph manifold regularizer.

**TABLE 5:** Classification performance of different methods.

**Method**	**ACC (%) ± STD**	**SEN (%) ± STD**	**SPE (%) ± STD**	**AUC ± STD**
PC (Jiang et al., 2019)	81.0570 ± 0.2551	77.2975 ± 0.3529	86.4165 ± 0.3682	0.8986 ± 0.0026
SR (Jiang et al., 2019)	73.9135 ± 0.2756	68.7518 ± 0.3423	79.0753 ± 0.5537	0.8237 ± 0.0009
MR (Li et al., 2020c)	49.8402 ± 1.1050	**96.3184 ± 5.3426**	3.3620 ± 4.3518	0.8291 ± 0.0404
SMR (Li et al., 2020c)	74.3410 ± 0.3876	68.9902 ± 0.4506	79.6918 ± 0.5397	0.8275 ± 0.0022
HMR	81.4570 ± 0.2727	76.6237 ± 0.3087	86.2903 ± 0.3670	0.9005 ± 0.0017
SHMR	**82.4946 ± 0.2827**	77.2473 ± 0.5747	**87.7419 ± 0.2286**	**0.9021 ± 0.0007**

### Discriminative Brain Regions

**FIGURE 4:**
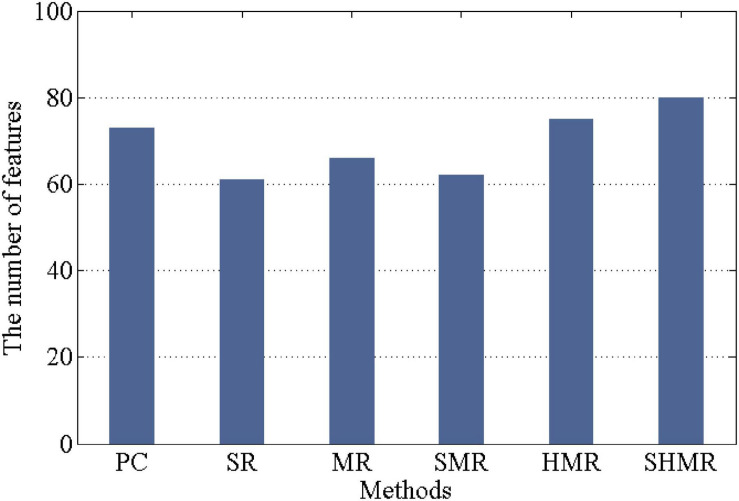
Number of features selected by different methods in 10-fold cross-validation.

In each 10-fold cross-validation, the number of selected features determines the quality of the DBFN. If the number of selected features is larger, the DBFN constructed by the corresponding method may contain more potential information. Therefore, in 10-fold cross-validation, we counted the number of selected features in different methods, that is, the number of selected weighted-graph local clustering coefficients, as shown in Figure 4. We can find that the SHMR method has more features selected in the 10-fold cross-validation than other methods, so the SHMR method can select more stable features.

**FIGURE 5:**
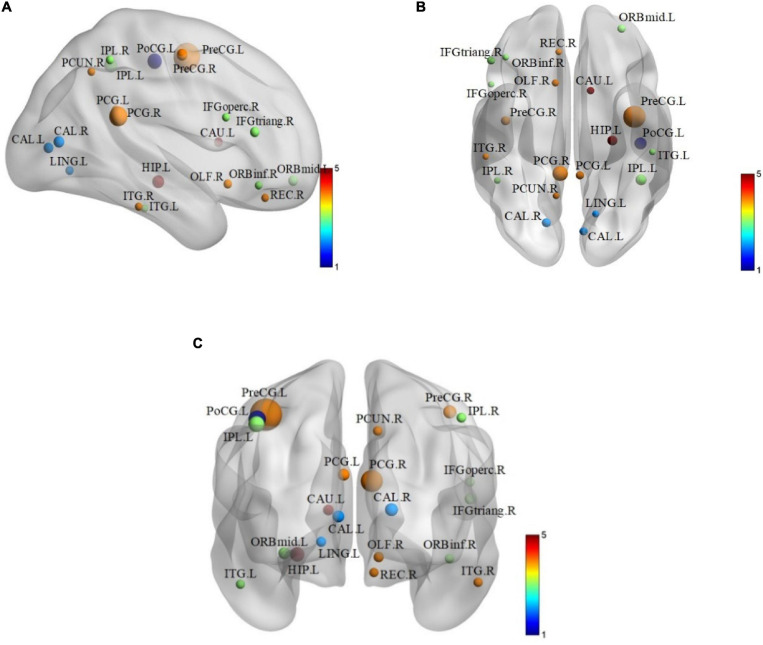
The layouts of discriminative brain regions. **(A)** Coronary figure. **(B)** Axis figure. **(C)** Sagittal figure.

In order to find some biomarkers for MCI diagnosis, we search for discriminative features and consider that features with higher frequency in 10-fold cross-validation are discriminative features. Therefore, we count features with high frequency in 10-fold cross-validation. There are 21 brain regions corresponding to these features, which are called discriminative brain regions. The details of the discriminative brain regions are shown in Table 6. Then we use the BrainNet Viewer toolbox^4^ (Xia et al., 2013) to visualize the discriminative brain regions. These discriminative brain regions are mapped to the ICBML52 template, and we use the JET template for color marking. The visualization results are shown in Figure 5.

**TABLE 6:** Discriminative brain regions.

**ID**	**Regions**	**Abbreviations (L:left R:right)**	**MNI coordinates**			**References**
			***X*(mm)**	***Y*(mm)**	***Z*(mm)**	
1	Precentral_L	PreCG.L	−38.65	−5.68	50.94	
2	Precentral_R	PreCG.R	41.37	−8.21	52.09	Zhang H. et al., 2016
9	Frontal_Mid_Orb_L	ORBmid.L	−30.65	50.43	−9.62	Zhang et al., 2018
12	Frontal_Inf_Oper_R	IFGoperc.R	50.20	14.98	21.41	Chen et al., 2016
14	Frontal_Inf_Tri_R	IFGtriang.R	50.33	30.16	14.17	Salvatore et al., 2015
16	Frontal_Inf_Orb_R	ORBinf.R	41.22	32.23	−11.91	Salvatore et al., 2015
22	Olfactory_R	OLF.R	10.43	15.91	−11.26	Sun et al., 2012
28	Rectus_R	REC.R	8.35	35.64	−18.04	
35	Cingulum_Post_L	PCG.L	−4.85	−42.92	24.67	Zhang et al., 2018
36	Cingulum_Post_R	PCG.R	7.44	−41.81	21.87	Wee et al., 2012
37	Hippocampus_L	HIP.L	−25.03	−20.74	−10.13	Salvatore et al., 2015
43	Calcarine_L	CAL.L	−7.14	−78.67	6.44	Xu et al., 2016
44	Calcarine_R	CAL.R	15.99	−73.15	9.40	
47	Lingual_L	LING.L	−14.62	−67.56	−4.63	
57	Postcentral_L	PoCG.L	−31.16	−40.30	−20.23	Xu et al., 2016
61	Parietal_Inf_L	IPL.L	−42.80	−45.82	46.74	
62	Parietal_Inf_R	IPL.R	46.46	−46.29	49.54	Salvatore et al., 2015
68	Precuneus_R	PCUN.R	9.98	−56.05	43.77	
71	Caudate_L	CAU.L	−11.46	11.00	9.24	Salvatore et al., 2015
89	Temporal_Inf_L	ITG.L	−49.77	−28.05	−23.17	Zhang et al., 2018
90	Temporal_Inf_R	ITG.R	53.69	−31.07	−22.32	

From Table 6 and Figure 5, we can find that some selected discriminative brain regions, including the left posterior cingulate gyrus (PCG.L), right posterior cingulate gyrus (PCG.R), left hippocampus (HIP.L), left inferior parietal, supramarginal, and angular gyri (IPL.L), right inferior parietal, supramarginal, and angular gyri (IPL.R), right precuneus (PCUN.R), left inferior temporal gyrus (ITG.L), and right inferior temporal gyrus (ITG.R), belong to the regions in the default mode network (DMN) (Bi et al., 2020a,b; Jiao et al., 2020). Most of the selected brain regions have been widely considered to be related to AD and MCI, which is consistent with the results of previous related research. Take the PCG.L, PCG.R, HIP.L, PCUN.R, ITG.L, and ITG.R as examples. Both PCG.L and PCG.R are involved in the formation of memory, and HIP.L is responsible for the storage, conversion, and orientation of long-term memory. PCUN.R is associated with many high-level cognitive functions, such as episodic memory, self-related information processing, and consciousness generation. ITG.L and ITG.R belong to the temporal lobe, which have the function of processing auditory information, and they are also related to memory and emotion. If ITG.L and ITG.R are damaged, it will cause personality changes. PCUN.R, ITG.L, and ITG.R demonstrate that DMN plays an important role in cognitive function and neuromodulation (Jiao et al., 2017a,b). In addition, some brain regions belonging to the prefrontal and occipital lobes are extracted, such as ORBmid.L, IFGoperc.R, and LING.L. It indicates that the language, vision, and motor perception of MCI patients have changed compared with people without MCI (Wee et al., 2011).

## Discussion

In recent years, researchers have shown an increased interest in the epidemiology, clinical characteristics, neuroimaging, biomarkers, mechanism of disease, neuropathology, and clinical trials of MCI. The challenges remain around the borders of the condition, i.e., between normal aging and early MCI and between MCI and clinical AD. However, with the development new neuroimaging techniques, these transitional states may be clarified. A major study indicates an annual rate of progression from cognitively healthy to the aMCI state of 3% per year. In addition, 26% of aMCI subjects have progressed to AD over 12 months, while another 4% of the aMCI subjects have reverted to a cognitively healthy status (Petrella and Doraiswamy, 2005). To date, relatively little research has been carried out on the MCI classification. Herein, our study proposes a DBFN construction method via HMR. We then apply this method to MCI classification. In this method, the DBFN construction method based on PC method is first transformed into an optimization model, and we construct SDBFNs by adding a hyper-graph manifold regularizer into the optimization model. The classification performance of SDBFNs for MCI patients and normal subjects outperforms other comparable methods.

Most research only considers the pair-wise relationship between brain regions and ignores the high-order relationship between multiple brain regions. This high-order relationship can also be regarded as the relationship between functional connections, which is important prior information. Nowadays, related research has explored this high-order relationship. For example, Chen et al. (2016) used correlation’s correlation to construct high-order functional networks, and reduced the dimensionality of high-order functional networks through k-means clustering method. The effectiveness of this method is verified in identifying MCI. Zhou et al. (2018) proposed a high-order functional network construction method based on matrix variate normal distribution (MVND). This method uses BFNs as samples and assumes that features in these samples follow MVND. Then, the maximum-likelihood estimation (MLE) for MVND is calculated to obtain the final high-order functional networks. However, these two methods have some shortcomings. The method of Chen et al. (2016) involves many parameters, which may easily lead to overfitting when the number of training data is limited, and this method is not supported by a mathematical model. The method of Zhou et al. (2018) requires strict assumptions before the subsequent conclusions can be established, so describing this complex relationship is very important. In a hyper-graph, a hyper-edge can connect more than two vertices, so the hyper-graph can naturally model this high-order relationship well.

However, our method also has issues which need to be improved. First, it is a very important step to construct the hyper-graph. Hence, we use the KNN method to construct the hyper-graph, which is not interpretable in the field of neuroimaging. Inspired by the work of Jie et al. (2016), we can use the SR method to construct the hyper-graph in future. Second, the main work of this study focuses on the DBFN construction method and we use the *t*-test method to select features. The improvement strategies for feature selection include simple improvement of feature selection method. The training set is combined with the test set to iteratively select the features which improve the classification performance step by step.

In summary, our method makes up for the problem that most methods for BFN construction cannot reflect the pair-wise relationship between multiple brain regions well. We apply this method to MCI classification, and have achieved the best classification ACC which outperforms the compared methods. Moreover, the discriminative brain regions obtained by our method can better reflect the pathogenic mechanism of MCI. Our future work will solve the following problems. First, we only classify Normal subjects and MCI subjects, and consider the binary problem. In the future, we can set up multi-class classifications, such as adding AD subjects to form a three-class problem and verifying our method. In addition, the dataset we used is relatively small, which may affect the promotion performance of the classifier. In practical applications, we will try to use other methods, such as transfer learning, to design specific methods for BFNs and further improve classification performance.

## Data Availability Statement

The original contributions presented in the study are included in the article/supplementary material. Further inquiries can be directed to the corresponding author/s.

## Ethics Statement

Ethical review and approval was not required for the study on human participants in accordance with the local legislation and institutional requirements. Written informed consent for participation was not required for this study in accordance with the national legislation and the institutional requirements.

## Author Contributions

ZJ, S-HW, and CW designed the research. YJ, YZ, and HS performed the study. YJ and HS analyzed the data. YJ wrote the manuscript. ZJ and CW revised the manuscript. All authors read and approved the final manuscript.

## Conflict of Interest

The authors declare that the research was conducted in the absence of any commercial or financial relationships that could be construed as a potential conflict of interest.

## Footnotes

1 http://www.nitrc.org/projects/modularbrain/

2 http://www.fil.ion.ucl.ac.uk/spm/

3 http://rfmri.org/dparsf/

4 https://www.nitrc.org/projects/bnv/

## References

[B1] Alzheimer’s Association, (2012). Alzheimer’s disease facts and figures. *Alzheimers Dement.* 8 131–168. 22404854 10.1016/j.jalz.2012.02.001

[B2] Basser P. J. Pierpaoli C. (2011). Microstructural and physiological features of tissues elucidated by quantitative-diffusion-tensor MRI. *J. Magn. Reson.* 213 560–570. 10.1016/j.jmr.2011.09.022 22152371

[B3] Bi X. A. Hu X. Wu H. Wang Y. (2020a). Multimodal data analysis of Alzheimer’s disease based on clustering evolutionary random forest. *IEEE J. Biomed. Health Inform.* 24 2973–2983. 10.1109/jbhi.2020.2973324 32071013

[B4] Bi X. A. Hu X. Xie Y. M. Wu H. (2021). A novel CERNNE approach for predicting Parkinson’s Disease-associated genes and brain regions based on multimodal imaging genetics data. *Med. Image Anal.* 67:101830. 10.1016/j.media.2020.101830 33096519

[B5] Bi X. A. Liu Y. C. Xie Y. M. Hu X. Jiang Q. H. (2020b). Morbigenous brain region and gene detection with a genetically evolved random neural network cluster approach in late mild cognitive impairment. *Bioinformatics* 36 2561–2568. 10.1093/bioinformatics/btz967 31971559 PMC7178433

[B6] Chang C. C. Lin C. J. (2011). LIBSVM: a library for support vector machines. *ACM Trans. Intell. Syst. Technol.* 2 389–396.

[B7] Chang C. T. Glover G. H. (2010). Time-frequency dynamics of resting-state brain connectivity measured with fMRI. *Neuroimage* 50 81–98. 10.1016/j.neuroimage.2009.12.011 20006716 PMC2827259

[B8] Chen X. B. Zhang H. Gao Y. Wee C. Y. Li G. Shen D. G. (2016). High-order resting-state functional connectivity network for MCI classification. *Hum. Brain Mapp.* 37 3282–3296. 10.1002/hbm.23240 27144538 PMC4980261

[B9] Elhamifar E. Vidal R. (2013). Sparse subspace clustering: algorithm, theory, and applications. *IEEE Trans. Pattern Anal. Mach. Intell.* 35 2765–2781. 10.1109/tpami.2013.57 24051734

[B10] Gauthier S. Reisberg B. Zaudig M. Petersen R. Ritchie K. Broich K. (2006). Mild cognitive impairment. *Lancet* 367 1262–1270. 16631882 10.1016/S0140-6736(06)68542-5

[B11] Jiang X. Zhang L. M. Qiao L. S. Shen D. G. (2019). Estimating functional connectivity networks via low-rank tensor approximation with applications to MCI identification. *IEEE Trans. Biomed. Eng.* 67 1912–1920. 31675312 10.1109/TBME.2019.2950712

[B12] Jiao Z. Q. Ji Y. X. Zhang J. H. Shi H. F. Wang C. (2020). Constructing dynamic functional networks via weighted regularization and tensor low-rank approximation for early mild cognitive impairment classification. *Front. Cell Dev. Biol.* 8:610569. 10.3389/fcell.2020.610569 33505965 PMC7829545

[B13] Jiao Z. Q. Wang H. Ma K. Zou L. Xiang J. B. (2017a). Directed connectivity of brain default networks in resting state using GCA and motif. *Front. Biosci. Landmark* 22:1634–1643. 10.2741/4562 28410136

[B14] Jiao Z. Q. Wang H. Ma K. Zou L. Xiang J. B. Wang S. H. (2017b). Effective connectivity in the default network using Granger causal analysis. *J. Med. Imaging Health Inform.* 7 407–415. 10.1166/jmihi.2017.2029

[B15] Jiao Z. Q. Xia Z. W. Ming X. L. Cheng C. Wang S. H. (2019). Multi-scale feature combination of brain functional network for eMCI classification. *IEEE Access* 7 74263–74273. 10.1109/access.2019.2920978

[B16] Jiao Z. Q. Zou L. Cao Y. Qian N. Ma Z. H. (2014). Effective connectivity analysis of fMRI data based on network motifs. *J. Supercomput.* 67 809–819.

[B17] Jie B. Wee C. Y. Shen D. G. Zhang D. Q. (2016). Hyper-connectivity of functional networks for brain disease diagnosis. *Med. Image Anal.* 32 84–100. 10.1016/j.media.2016.03.003 27060621 PMC5333488

[B18] Jung T. P. Makeig S. Humphries C. Lee T. W. Mckeown M. J. Iragui V. (2000). Removing electroencephalographic artifacts by blind source separation. *Psychophysiology* 37 163–178. 10.1111/1469-8986.3720163 10731767

[B19] Li W. K. Geng C. X. Chen S. C. (2020a). Leave zero out: towards a no-cross-validation approach for model selection. *arXiv* [preprint] arXiv:2012.13309

[B20] Li W. K. Qiao L. S. Shen D. G. (2020b). Towards a better estimation of functional brain network for MCI identification: a transfer learning view. *IEEE J. Biomed. Health Inform.* 24 1160–11 10.1109/jbhi.2019.2934230 31403449 PMC7285887

[B21] Li W. K. Wang Z. X. Qiao L. S. Shen D. G. (2019). Functional brain network estimation with time series self-scrubbing. *IEEE J. Biomed. Health Inform.* 23 2494–2504. 10.1109/jbhi.2019.2893880 30668484 PMC6904893

[B22] Li W. K. Wang Z. X. Zhang L. M. Qiao L. S. Shen D. G. (2017). Remodeling pearson’s correlation for functional brain network estimation and autism spectrum disorder identification. *Front. Neuroinform.* 11:55. 10.3389/fninf.2017.00055 28912708 PMC5583214

[B23] Li W. K. Xu X. W. Jiang W. Wang P. J. Gao X. (2020c). Functional connectivity network estimation with an inter-similarity prior for mild cognitive impairment classification. *Aging* 12:17328. 10.18632/aging.103719 32921634 PMC7521542

[B24] Li Y. Liu J. Y. Gao X. Q. Jie B. Minjeong K. Pew-Thian Y. (2018). Multimodal hyper-connectivity of functional networks using functionally-weighted LASSO for MCI classification. *Med. Image Anal.* 52 80–96. 10.1016/j.media.2018.11.006 30472348

[B25] Lu S. Y. Lu Z. H. Zhang Y. D. (2019). Pathological brain detection based on AlexNet and transfer learning. *J. Comput. Sci.* 30 41–47. 10.1016/j.jocs.2018.11.008

[B26] Mourik J. E. M. Lubberink M. Schuitemaker A. Tolboom N. Boellaard R. (2009). Image-derived input functions for PET brain studies. *Eur. J. Nuclear Med. Mol. Imaging* 36 463–471. 10.1007/s00259-008-0986-8 19030855

[B27] Muldoon S. F. Bassett D. S. (2016). Network and multilayer network approaches to understanding human brain dynamics. *Philos. Sci.* 83 710–720. 10.1086/687857

[B28] Ou J. L. Lian Z. C. Xie L. Xiang L. Wang P. Hao Y. (2015). Atomic dynamic functional interaction patterns for characterization of ADHD. *Hum. Brain Mapp.* 35 5262–5278. 10.1002/hbm.22548 24861961 PMC6869011

[B29] Petrella J. R. Doraiswamy P. M. (2005). Alzheimer’s disease: 100 years of progress. *Neuroimaging Clin. N. Am.* 15 13–14.

[B30] Qiao L. S. Zhang H. Kim M. J. Teng S. H. Zhang L. M. Shen D. G. (2016). Estimating functional brain networks by incorporating a modularity prior. *Neuroimage* 141 399–407. 10.1016/j.neuroimage.2016.07.058 27485752 PMC5338311

[B31] Roebroeck A. Formisano E. Goebel R. (2005). Mapping directed influence over the brain using Granger causality and fMRI. *Neuroimage* 25 230–242. 10.1016/j.neuroimage.2004.11.017 15734358

[B32] Rubinov M. Sporns O. (2010). Complex network measures of brain connectivity: uses and interpretations. *Neuroimage* 52 1059–1069. 10.1016/j.neuroimage.2009.10.003 19819337

[B33] Salvatore C. Cerasa A. Battista P. Gilardi M. C. Quattrone A. Castiglioni I. (2015). Magnetic resonance imaging biomarkers for the early diagnosis of Alzheimer’s disease: a machine learning approach. *Front. Neurosci.* 9:307. 10.3389/fnins.2015.00307 26388719 PMC4555016

[B34] Shao W. Peng Y. Zu C. Wang M. L. Zhang D. Q. the Alzheimer’s Disease Neuroimaging Initiative (2019). Hypergraph based multi-task feature selection for multimodal classification of Alzheimer’s disease. *Comput. Med. Imaging Graph.* 80:101663. 10.1016/j.compmedimag.2019.101663 31923610

[B35] Smythies L. E. Sellers M. Clements R. H. Mosteller-Barnum M. Meng G. Benjamin W. H. (2005). Human intestinal macrophages display profound inflammatory anergy despite avid phagocytic and bacteriocidal activity. *J. Clin. Invest.* 115 66–75. 10.1172/jci200519229 15630445 PMC539188

[B36] Sun G. H. Raji C. A. MacEachern M. P. Burke J. F. (2012). Olfactory identification testing as a predictor of the development of Alzheimer’s dementia: a systematic review. *Laryngoscope* 122 1455–1462. 10.1002/lary.23365 22552846

[B37] Tobia M. J. Hayashi K. Ballard G. Gotlib I. H. Waugh C. E. (2017). Dynamic functional connectivity and individual differences in emotions during social stress. *Hum. Brain Mapp.* 38 6185–6205. 10.1002/hbm.23821 28940859 PMC6866845

[B38] Tzourio-Mazoyer N. Landeau B. Papathanassiou D. Crivello F. Etard O. Delcroix N. (2002). Automated anatomical labeling of activations in SPM using a macroscopic anatomical parcellation of the MNI MRI single-subject brain. *Neuroimage* 15 273–289. 10.1006/nimg.2001.0978 11771995

[B39] Wee C. Y. Yap P. T. Li W. B. Kevin D. Jeffrey N. B. Guy G. P. (2011). Enriched white matter connectivity networks for accurate identification of MCI patients. *Neuroimage* 54 1812–1822. 10.1016/j.neuroimage.2010.10.026 20970508 PMC3008336

[B40] Wee C. Y. Yap P. T. Zhang D. Q. Denny K. Browndyke J. N. Potter G. G. (2012). Identification of MCI individuals using structural and functional connectivity networks. *Neuroimage* 59 2045–2056. 10.1016/j.neuroimage.2011.10.015 22019883 PMC3254811

[B41] Wee C. Y. Yap P. T. Zhang D. Q. Wang L. H. Shen D. G. (2014). Group-constrained sparse fMRI connectivity modeling for mild cognitive impairment identification. *Brain Struct. Funct.* 219 641–656. 10.1007/s00429-013-0524-8 23468090 PMC3710527

[B42] Xia M. R. Wang J. H. He Y. (2013). BrainNet Viewer: a network visualization tool for human brain connectomics. *PLoS One* 8:e68910. 10.1371/journal.pone.0068910 23861951 PMC3701683

[B43] Xu L. L. Wu X. Li R. Chen K. W. Long Z. Y. Zhang J. C. (2016). Prediction of Progressive Mild Cognitive Impairment by multi-modal neuroimaging biomarkers. *J. Alzheimers Dis.* 51 1045–1056. 10.3233/jad-151010 26923024

[B44] Xu X. W. Li W. K. Mei J. Tao M. L. Wang X. B. Zhao Q. H. (2020). Feature selection and combination of information in the functional brain connectome for discrimination of Mild Cognitive Impairment and analysis of altered brain patterns. *Front. Aging Neurosci.* 12:28. 10.3389/fnagi.2020.00028 32140102 PMC7042199

[B45] Xue Y. F. Zhang L. M. Qiao L. S. Shen D. G. (2020). Estimating sparse functional brain networks with spatial constraints for MCI identification. *PLoS One* 15:e0235039. 10.1371/journal.pone.0235039 32707574 PMC7381102

[B46] Yan C. G. Cheung B. Kelly C. Colcombe S. Cameron Craddock R. Martino A. D. (2013). A comprehensive assessment of regional variation in the impact of head micromovements on functional connectomics. *Neuroimage* 76 183–201. 10.1016/j.neuroimage.2013.03.004 23499792 PMC3896129

[B47] Yu J. Rui Y. Tang Y. Y. Tao D. C. (2014). High-order distance-based multiview stochastic learning in image classification. *IEEE Trans. Cybern.* 44 2431–2442. 10.1109/tcyb.2014.2307862 25415948

[B48] Zhang H. Chen X. B. Shi F. Gang L. Kim M. J. Giannakopoulos P. (2016). Topographical information-based high-order functional connectivity and its application in abnormality detection for Mild Cognitive Impairment. *J. Alzheimers Dis.* 54 1095–1112. 10.3233/jad-160092 27567817 PMC5437847

[B49] Zhang Y. Zhang H. Chen X. B. Liu M. X. Zhu X. F. Lee S. W. (2018). Strength and similarity guided group-level brain functional network construction for MCI diagnosis. *Pattern Recogn.* 88 421–430. 10.1016/j.patcog.2018.12.001 31579344 PMC6774624

[B50] Zhang Y. D. Dong Z. C. Ji G. L. Wang S. H. (2015a). Effect of spider-web-plot in MR brain image classification. *Pattern Recogn. Lett.* 62 14–16. 10.1016/j.patrec.2015.04.016

[B51] Zhang Y. D. Dong Z. C. Liu A. J. Wang S. H. Ji G. L. (2015b). Magnetic resonance brain image classification via stationary wavelet transform and generalized eigenvalue proximal support vector machine. *Med. Imaging Health Inform.* 5 1395–1403. 10.1166/jmihi.2015.1542

[B52] Zhang Y. D. Wang S. H. Phillips P. Yang J. Yuan T. F. (2016). Three-Dimensional eigenbrain for the detection of subjects and brain regions related with Alzheimer’s disease. *J. Alzheimers Dis.* 50 1163–1179. 10.3233/jad-150988 26836190

[B53] Zhou D. Huang J. Scholkopf B. (2007). “Learning with hypergraphs: clustering, classification, and embedding,” in *Proceedings of the 19th International Conference on Neural Information Processing Systems*, Vancouver, BC, 1601–1608.

[B54] Zhou Y. Y. Qiao L. S. Li W. K. Zhang L. M. Shen D. G. (2018). Simultaneous estimation of low- and high-order functional connectivity for identifying mild cognitive impairment. *Front. Neuroinform.* 12:3. 10.3389/fninf.2018.00003 29467643 PMC5808180

[B55] Zien J. Y. Schlag M. D. F. Chan P. K. (1999). Multilevel spectral hypergraph partitioning with arbitrary vertex sizes. *IEEE Trans. Comput. Aided Design Integr. Circuits Syst.* 18 1389–1399. 10.1109/43.784130

